# Role of Hospital Connectedness in Brain Metastasis Outcomes

**DOI:** 10.1001/jamanetworkopen.2024.35051

**Published:** 2024-09-23

**Authors:** Lilin Tong, Ruchit V. Patel, Ayal A. Aizer, Amar Dhand, Wenya Linda Bi

**Affiliations:** 1 Department of Neurosurgery, Brigham and Women’s Hospital, Boston, Massachusetts; 2 Boston University Aram V. Chobanian & Edward Avedisian School of Medicine, Boston, Massachusetts; 3 Harvard Medical School, Boston, Massachusetts; 4 Department of Radiation Oncology, Brigham and Women’s Hospital, Boston, Massachusetts; 5 Department of Neurology, Brigham and Women’s Hospital, Boston, Massachusetts

## Abstract

**Importance:**

Although patients with brain metastases receive interdisciplinary and multi-institutional care, the association between neuro-oncologic care networks and patient outcomes remains unknown. As patients often interact with multiple facilities, quantifying this association across a network of hospitals is critical to capture the complexity of the health care journey for patients with brain metastases.

**Objective:**

To evaluate how statewide health care network metrics are associated with inpatient mortality and hospital length of stay (LOS) for patients with brain metastases.

**Design, Setting, and Participants:**

This multicenter, statewide cohort study used data from the 2018 to 2019 Healthcare Cost and Utilization Project State Inpatient and Emergency Department Databases. Primary analyses were completed by August 2023. Participants included adults with a brain metastases receiving care in Massachusetts.

**Exposure:**

All inpatient and emergency department visits mapped for patients following the first diagnosis of brain metastasis.

**Main Outcomes and Measures:**

Inpatient mortality and hospital LOS were the main outcomes assessed. Hospital interdependence in brain metastases care was calculated using a connectedness score (weighted degree: weighted sum of ties to other care facilities). The association between hospital connectedness and clinical outcomes was analyzed using mixed-effects logistic and linear regression models, adjusting for hospital-level features.

**Results:**

In this cohort study, 4679 patients with brain metastases were identified with inpatient or ED encounters in Massachusetts (from 2018 to 2019). The median (IQR) age was 64 (57-73) years, and 2559 (55%) were female. There was interdependence in brain metastases care, with 993 patients (21%) visiting 2 or more unique hospitals. Highly connected hospitals were heterogeneous, with many being small and one-half lacking subspecialty neuro-oncologic care or teaching status. Increased hospital connectedness was significantly associated with improved inpatient mortality for patients with brain metastases, with the lowest connectedness quartile associated with more than double the risk of mortality compared with the highest quartile (odds ratio, 2.34; 95% CI, 1.33-4.11; *P* = .003). A stepwise increase in inpatient mortality risk was observed as hospital connectedness decreased, independently of hospital volume. Furthermore, intermediate hospital connectedness was associated with increased hospital LOS (coefficient, 1.08; 95% CI, 0.17-1.95; *P* = .006).

**Conclusions and Relevance:**

This study found that hospital-to-hospital interconnectedness was significantly associated with improved clinical outcomes for patients with brain metastases. The salience of network metrics highlights their potential role alongside other patient-level and hospital-level variables to evaluate and improve oncology care delivery.

## Key Points

**Question:**

Is hospital connectedness associated with clinical outcomes of patients with brain metastases?

**Findings:**

In this cohort study of 4679 patients with brain metastases in Massachusetts, increased connectedness of a hospital, defined as the weighted sum of ties to other care facilities, was associated with improved inpatient mortality. Hospital connectedness operated independently from hospital volume in its association with inpatient mortality and length of stay.

**Meaning:**

These findings suggest that relationships between hospitals are associated with changes in patient outcomes in the interdisciplinary care of patients with brain metastases, likely owing to specialized and interdisciplinary care required in the disease management.

## Teaser

This cohort study examines how statewide health care network metrics are associated with inpatient mortality and hospital length of stay for adults patients with brain metastases receiving care in Massachusetts.

## Introduction

The treatment of patients with brain metastasis is increasingly interdisciplinary.^1^ With therapeutic advances, these once largely palliative conditions are seeing prolonged survival and require longer-term management involving multiple specialists, including neurosurgeons, radiation oncologists, and medical oncologists, across various care delivery settings.^2^ Patients require intensive treatments and large range of medical services and often travel between facilities at different points of their disease course to receive specific care.^3^ As such, the quality of the care received may reflect the network of hospitals with which patients interact and the relationships among care facilities.^4^

The association between multidimensional hospital networks and clinical outcome is underappreciated in the literature.^4,5^ Prior studies^6,7^ on brain tumor health delivery often treat hospitals in isolation, citing improved outcome with high-volume or academic centers without capturing the role of other important care facilities, such as community hospitals. These analyses may deemphasize the role of community hospitals, which can provide important services in closer proximity to patients’ residences. Local facilities can facilitate ease of access to frequently needed treatments and testing and allow for closer patient monitoring.^8^ To study the role of a hospital’s position in an integrated clinical network, we sought to use measures of connectedness to map a statewide hospital network for patients with brain metastasis requiring inpatient care. Our analysis examines how hospital connectedness may be associated with mortality and hospital length of stay (LOS), underscoring how network approaches can complement our understanding of factors influencing effective care delivery.

## Methods

### Patient Population

We conducted a cohort study using the Healthcare Cost and Utilization Project (HCUP) State Emergency Department Databases and State Inpatient Databases for Massachusetts from 2018 to 2019.^9,10^ HCUP is maintained by the Agency for Healthcare Research and Quality delivery, which manages state and national inpatient and emergency department discharge data. We further incorporated data from the American Hospital Association Annual Survey of Hospitals to capture inpatient facility characteristics.^11^ HCUP datasets are limited and not subject to institutional review board approval under the Health Insurance Portability and Accountability Act. Individual patients are not identifiable, and all analytical methods were performed along HCUP data use agreement guidelines. This report follows the Strengthening the Reporting of Observational Studies in Epidemiology (STROBE) reporting guidelines for observational studies.

We used *International Statistical Classification of Diseases, Tenth Revision, Clinical Modification* codes corresponding to a brain metastasis diagnosis (C79.31 and C79.32) to extract patients with at least 1 recorded claim over the study period. A deidentified patient identifier and timing variable were used to link patient claims and order their encounters chronologically. Patient encounters were included after the first recorded brain metastases diagnosis to ensure that all included encounters occurred in the setting of an existing brain metastases diagnosis.

### Network Generation

We created directed networks using all included encounters following brain metastasis diagnosis to understand patient sharing dynamics. Each hospital that received a patient with brain metastasis served as a network node, whereas network links represent patients who were seen one hospital and subsequently another hospital. Movement from one hospital to another reflected transfers, physician-referred care, and patient-determined care. Directionality was established by determining patient movement into a facility vs out of a facility. A net degree of patient flow was established by subtracting the total number of patients coming in vs out of a facility. Network generation and modeling were performed in R statistical software version 4.2.1 (R Project for Statistical Computing).

Each hospital was assigned a connectedness score (also known as weighted degree), defined as the sum of all patients shared with other hospitals in the network.^12^ As the connectedness score of a hospital increases, the number and strength of its ties to other hospitals also increases.^13^ Depending on their connectedness score, each hospital was assigned a connectedness quartile, ranging from least connected (quartile 1) to most connected (quartile 4). Hospital case volume for patients with brain metastases was also quantified, defined as the total number of brain metastasis cases seen by that facility over the data years. Hospital volume was also divided into quartiles, ranging from the lowest volume (quartile 1) to highest volume (quartile 4). Net patient flow was determined for each hospital, which is defined as the sum of patients received by a given hospital subtracted by the sum of patients sent to another hospital; a positive value indicates net inward patient flow, whereas a negative value indicates net outward patient flow. To determine patient characteristics such as age, race, and income, only the data from the first row of each patient’s visit set was used. Any data where the number of patients is 10 or fewer were not reported per HCUP data use agreement. Data on race are reported to identify potential social inequities that may influence health outcomes.

### Statistical Analysis

We compared hospital-level and patient-level characteristics of hospitals of different connectedness scores. Continuous and categorical baseline characteristics among the quartiles were compared using the analysis of variance and χ^2^ test, respectively, with post hoc Tukey honestly significant difference test or Holm adjustment. We used mixed-effects multivariable regression models to determine the association between hospital connectedness and all-cause in-hospital mortality (logistic regression), as well as hospital LOS (linear regression). Given that multiple hospital admissions were nested within hospitals, we accounted for this clustering by including hospitals as a random effect in our model. Regression was adjusted with hospital-level factors, including teaching status, bed size, and age and comorbidity score (calculated using Elixhauser Comorbidity Index) of patients with brain metastasis. If patients were directly transferred between 2 hospital facilities with no gap in their care, inpatient LOS was considered the sum of LOS for both the sending and the receiving hospitalizations. Direct transfer is defined as a patient being discharged from a hospital and admitted to another within 1 day. This definition aligns with existing literature, which commonly indicates that transfers typically occur within 4 to 6 hours of discharge, and similar methods have been used in previous studies.^3,14^ The highest quartile (quartile 4) was the reference level for analysis for hospital connectedness and hospital volume. The mixed-effect multivariable models controlled for hospital-level characteristics (ie, bed size, teaching status, hospital case volume, age of patients with brain metastases at initial presentation encountered by hospital, and Elixhauser Comorbidity index encountered by hospital). All statistical tests were performed in R statistical software version 4.2.1 (R Project for Statistical Computing) with a significance level of 2-tailed *P* < .05. We conducted most of the analysis by August 2023.

## Results

We identified 4679 patients with brain metastases across Massachusetts, with at least 1 inpatient or emergency department encounter. The median (IQR) age of patients was 64 (57-73) years, and 2559 (55%) were female. Patients visited 55 unique facilities, which varied in size, clinical capabilities, teaching status, and connectedness to each other.

Stratifying hospitals caring for patients with brain metastases into quartiles by their connectedness score revealed heterogeneity in hospital characteristics among the most highly connected hospitals (quartile 4) (Table). Highly connected hospitals demonstrated a bimodal distribution of bed size, with many possessing bed numbers comparable to those of less-connected facilities (125-325 beds), although their mean bed size was larger (Table). Fifty percent of highly connected hospitals were not of teaching status or offered neuro-oncology, although they were enriched for neurosurgery and radiation oncology services compared with less-connected hospitals. Connectedness quartiles were further associated with regional location, with a greater proportion of highly connected hospitals localized in urban areas, whereas more than one-third of less-connected facilities were located in suburban areas. Socioeconomic factors also varied across connectedness quartiles, with patients presenting to highly connected hospitals more likely to have private insurance, higher income, and/or reside in larger metropolitan areas. Notably, hospitals in connectedness quartiles 3 and 4 had patients with similar comorbidity levels, which were significantly higher than those in quartiles 1 and 2, where patients generally had a lower comorbidity burden.

**Table.** Hospital and Patient Characteristics by Hospital Connectedness Quartile

Characteristic	Hospitals or patients, No. (%)^a^				*P* value^b^
	Quartile 1	Quartile 2	Quartile 3	Quartile 4	
Hospitals					
No. of beds					
Mean (SD)	116 (102)	150 (52)	256 (181)	436 (272)	<.001
Median (IQR)	90 (29-179)	154 (103-184)	216 (155-287)	334 (259-678)	
Teaching status	1 (7)	2 (14)	3 (23)	7 (50)	<.001
Hospital capabilities					
Neurosurgery	3 (21)	6 (43)	7 (54)	11 (79)	<.001
Radiation oncology	6 (43)	9 (64)	10 (77)	13 (93)	<.001
Neuro-oncology	2 (14)	2 (14)	4 (31)	7 (50)	<.001
Community type					
Developing suburban	5 (36)	1 (7)	2 (15)	0	<.001
Matured suburban	1 (7)	2 (14)	2 (15)	2 (14)	
Regional urban	6 (43)	9 (64)	6 (46)	6 (43)	
Core urban	2 (14)	2 (14)	3 (23)	6 (43)	
Patients					
Elixhauser Comorbidity Score					
Mean (SD)	28.7 (13.5)	28.8 (14.4)	30.1 (14.1)	31.6 (12.8)	<.001
Median (IQR)	29 (23-37)	28 (31-37)	31 (24-40)	29 (23-38)	
Age, y					
Mean (SD)	68.8 (12.4)	67.2 (10.8)	65.5 (11.9)	63.6 (12.6)	<.001
Median (IQR)	70 (61-78)	67 (60-74)	66 (58-73)	64 (57-72)	
Sex					
Female	134 (52)	231 (52)	513 (52)	1681 (56)	.03
Male	124 (48)	218 (48)	481 (48)	1302 (44)	
Payer					
Medicare	166 (64)	291 (66)	619 (62)	1590 (53)	<.001
Medicaid	24 (9)	47 (11)	115 (12)	230 (8)	
Private	60 (23)	96 (22)	191 (19)	1061 (36)	
Self-pay	NR^c^	NR^c^	30 (3)	23 (1)	
Other	NR^c^	NR^c^	39 (4)	79 (3)	
Race and ethnicity					
Asian or Pacific Islander	NR^c^	NR^c^	34 (3)	87 (3)	<.001
Black	NR^c^	12 (3)	71 (7)	127 (4)	
Hispanic	11 (4)	23 (5)	37 (4)	108 (4)	
White	231 (90)	397 (87)	822 (83)	2558 (86)	
Other^d^	NR^c^	NR^c^	12 (1)	41 (1)	
Income quartile by zip code					
Quartile 4	116 (45)	145 (33)	438 (44)	1634 (55)	<.001
Quartile 3	72 (28)	124 (28)	248 (25)	789 (26)	
Quartile 2	34 (13)	114 (26)	168 (17)	313 (11)	
Quartile 1	28 (11)	47 (11)	124 (12)	209 (7)	

^a^ Table shows comparison of the characteristics of hospitals as segregated into 4 quartiles (1-4) based on their connectedness score and the patients with brain metastases who received care as an inpatient or in the emergency department at these hospitals.

^b^ *P* < .05 represents any significant difference between the 4 connectedness quartiles for each variable.

^c^ NR (not reported) indicates values that cannot be reported per Healthcare Cost and Utilization Project data use agreement (ie, 10 or fewer patient-level observations) owing to risks of identification.

^d^ As defined by the Healthcare Cost and Utilization Project, the Other category characterizes a person who does not self-identify with any of the listed race categories.

**Figure 1.**
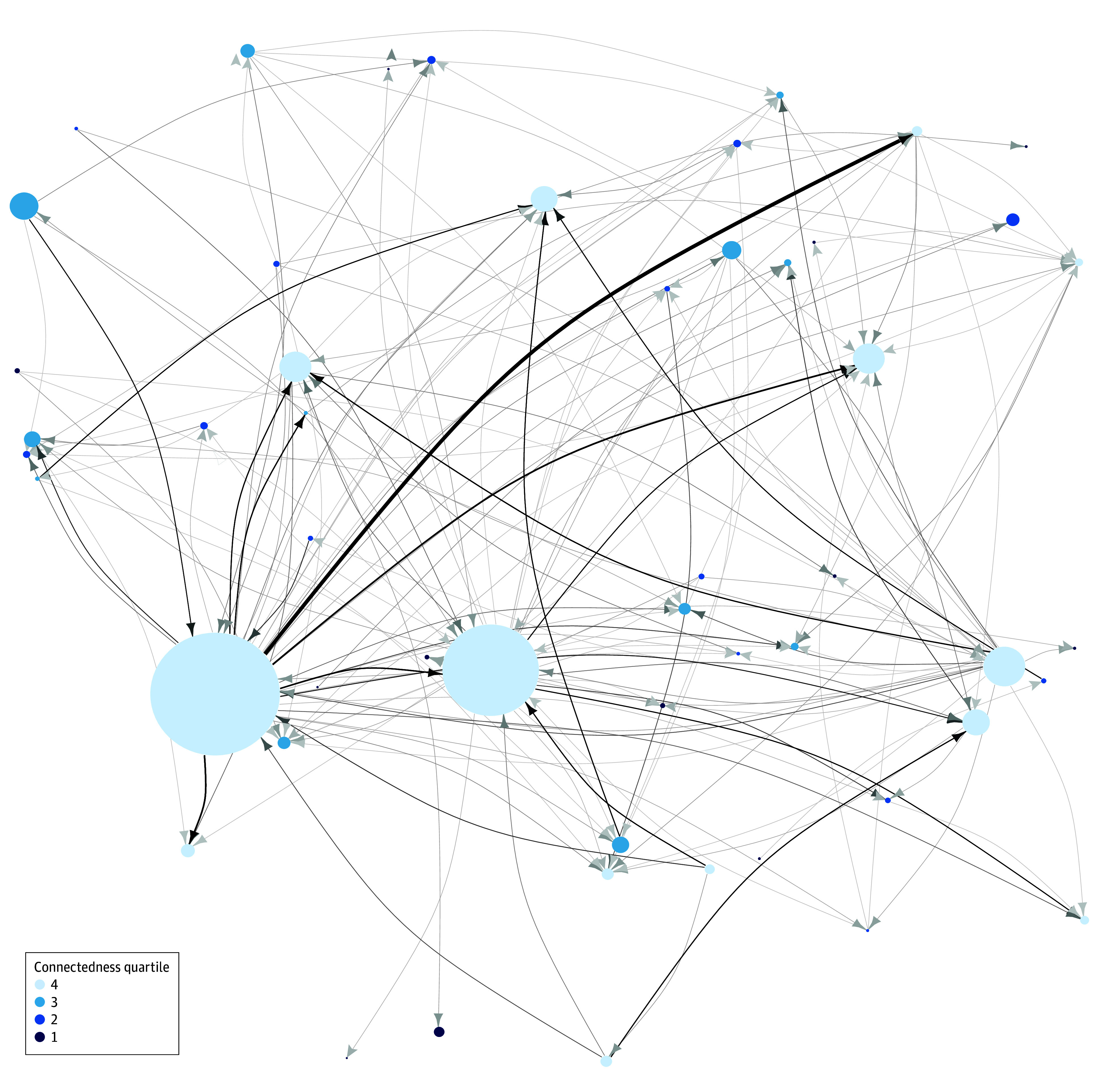
Interhospital Network for Patients With Brain Metastasis Figure shows a map of interhospital connections for brain metastasis in Massachusetts, where circles represent individual hospitals, size of the circle represents the number of annual inpatient or emergency department visits for patients with brain metastasis, and color represents connectedness quartile as demonstrated in the legend. Lines represent patients who were seen at one hospital and subsequently another hospital. Thickness of the line denotes frequency of a specific connection.

**Figure 2.**
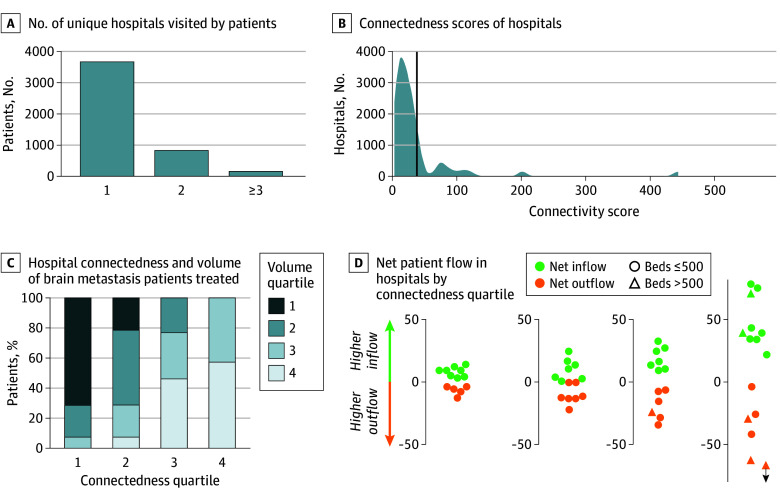
Network Dynamics and Patient Flow A, Number of unique hospitals visited by patients with brain metastasis as an inpatient or emergency department encounter in 2018 to 2019. B, Histogram of connectedness scores, also known as weighted degree, defined as the sum of all patients shared with other hospitals in the network. C, Association between hospital connectedness and volume of patients with brain metastasis treated at a given hospital, as stratified into quartiles for each hospital metric. D, Net patient flow for hospitals stratified by connectedness quartile.

Although several large nodes are present in the brain metastases care network, corresponding to large referral centers, numerous connections between facilities bypassed these nodes (Figure 1). In total, 993 patients (21%) received inpatient or ED care at 2 or more unique hospitals during this 2-year interval (Figure 2A). The connectedness score of individual hospitals varied: most hospitals clustered around a median (IQR) score of 20.0 (9.5-33.5), with some reaching connectedness scores of 100, 200, and greater than 400 (Figure 2B). However, highly connected hospitals were not synonymous with high-volume hospitals, with only 8 highly connected hospitals (57%) (quartile 4 connectedness) being of the highest volume facilities (quartile 4 volume) (Figure 2C).

Across all connectedness quartiles, hospitals exhibited a similar distribution of net patient flow into vs out of the facility. In the highest connectedness quartile, 8 hospitals (57%) had a net inflow of patients, and 6 (43%) had a net outflow (Figure 2D). Notably, even hospitals with larger bed sizes (ie, >500 beds) were evenly distributed between net inflow and outflow. Interestingly, our analysis did not show a difference in in-hospital mortality and LOS between hospitals with high inflow and those with high outflow, even after adjusting for hospital characteristics (eFigure 1 in Supplement 1).

**Figure 3.**
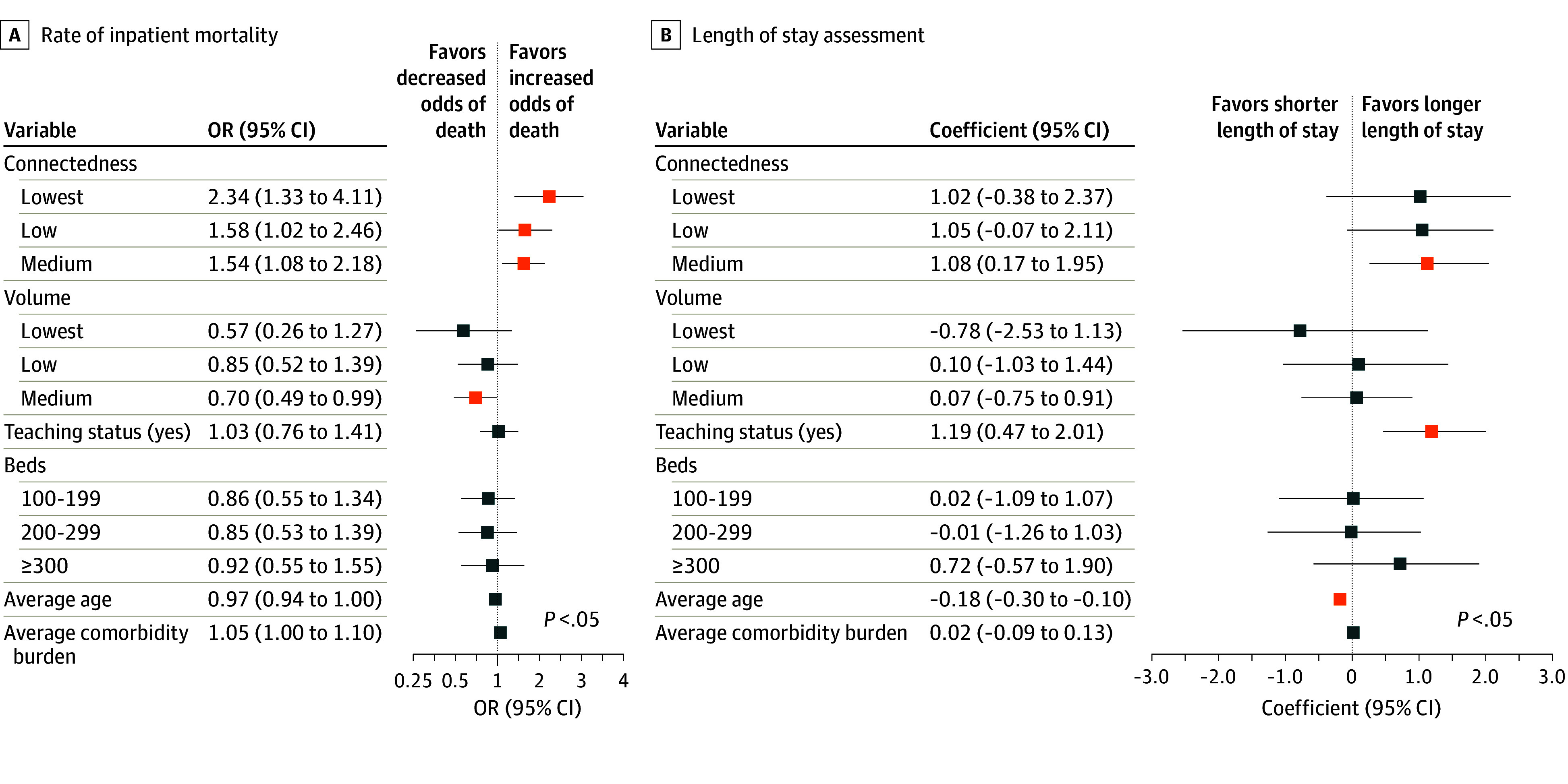
Hospital Connectedness and Volume of Patients With Brain Associated With Inpatient Mortality and Length of Stay The association of hospital connectedness and hospital volume with rate of inpatient mortality (A) and length of stay (B) displayed on a log scale were assessed, adjusted for teaching status, bed size, age, and comorbidity burden (calculated by Elixhauser Comorbidity Index). OR indicates odds ratio.

Multivariable-adjusted analysis using a mixed-effects model combining patient and hospital characteristics demonstrated that lower hospital connectedness was associated with a higher risk of inpatient mortality in a stepwise fashion (Figure 3A). Compared with the highest connectedness quartile, the lowest connectedness scores (quartile 1) was associated with an inpatient mortality odds ratio (OR) of 2.34 (95% CI, 1.33 to 4.11; *P* = .003). Connectedness quartile 2 had an OR for mortality of 1.58 (95% CI, 1.02 to 2.46; *P* = .04), whereas connectedness quartile 3 had an OR of 1.54 (95% CI, 1.08 to 2.18; *P* = .02). Conversely, hospital volume did not exhibit a similar association with mortality, although medium-volume hospital were associated with a lower risk of mortality compared with higher-volume hospitals (OR, 0.70; 95% CI, 0.49 to 0.99; *P* = .046). A similar stepwise pattern was observed with hospice discharges: less-connected hospitals were more likely to discharge patients to hospice (eFigure 2 in Supplement 1). Compared with inpatient mortality, hospital LOS had a differential association with hospital connectedness and hospital volume. Hospital connectedness was more variable in its association with LOS, with intermediate connectedness scores (quartile 3) reaching statistical significance for greater inpatient LOS (coefficient, 1.08; 95% CI, 0.17 to 1.95; *P* = .006) (Figure 3B). Hospital volume was similarly variable and was not significantly associated with changes in inpatient LOS after multivariable adjustment. Additional factors linked to hospital LOS emerged, including teaching status designation associated with longer LOS (coefficient, 1.19; 95% CI, 0.47 to 2.01; *P* = .01) and younger age associated with shorter LOS (coefficient, −0.18; 95% CI, −0.3 to −0.1; *P* < .001).

## Discussion

Brain metastasis is a disease requiring highly multidisciplinary, clinically integrated care, for which patients often navigate a range of medical facilities throughout their care journey.^1^ For many patients, inpatient encounters form a critical component of that care arc, from managing brain metastasis itself to primary and secondary complications associated with their illness.^15^ In this cohort study, we present some of the first evidence in brain tumor management demonstrating that hospital-to-hospital connections are associated with changes in clinical outcomes among patients with brain metastasis requiring inpatient care. Modeling the flow of patients on a statewide level demonstrates how a network approach can reveal quality metrics for individual hospitals that serve as a useful tool to enhance care delivery.

Although hospital resources and volume have traditionally been used to assess the clinical potential of hospital facilities, our findings show that connectedness is independent of these metrics. Indeed, approximately one-half of highly connected (quartile 4) hospitals provide care to only a moderate number of patients with brain metastases annually, and many of these hospitals lack in-house capabilities such as neurosurgery. Highly connected hospitals also did not attain their high connectedness score solely by receiving or transferring patients. There was an even distribution of hospitals with inward vs outward flow, even among large, highly connected hospitals. This is surprising, as one might expect larger hospitals to predominantly serve as receiving facilities. Conversely, some smaller hospitals with high connectedness scores accepted more patients than they transferred out. This suggests that smaller hospitals not only escalate patient care by transferring to larger facilities but also play a crucial role in deescalating care, with patients moving to smaller community hospitals after visiting larger ones, providing additional options, flexibility, and convenience for patient care. It is also worth noting that quality-of-care metrics did not differ between hospitals predominantly receiving patients and those primarily sending patients. This finding suggests that the benefits of an interconnected network are shared among all hospitals, rather than concentrated in a subset.

We found that lower hospital connectedness was highly associated with increased risk of mortality among inpatients with brain metastases, a trend that has been validated in other disease areas.^5^ Mechanistically, hospital connectedness may enable rapid escalation to subspecialty services when clinically indicated, while addressing resource strain, all of which can impact outcomes.^5^ Furthermore, we found a U-shaped association between hospital connectedness and LOS, where hospitals of connectedness quartile 3 showed longer LOS compared with their more-connected and less-connected counterparts. This finding reflects results from a prior study,^5^ which also reported a U-shaped distribution of outcomes based on hospital connectedness quartiles. This pattern may be due to hospitals in this quartile caring for patients with major comorbidities similar to those in highly connected hospitals, but lacking the network to offload complex cases to more central hospitals with potentially better capabilities. We did not find hospital volume to be associated with LOS. The emergence of an association between hospital connectedness with inpatient mortality and LOS, independently of traditional metrics such as hospital volume, suggests connectedness that may capture aspects of a patient’s care not readily apparent in isolated hospital analyses. This highlights connectedness as a novel method for assessing quality of care in an increasingly intertwined health care environment.

### Limitations

This study has limitations that should be mentioned. As a retrospective administrative database, HCUP is subject to variability in data entry and lacks annotation on specificity and severity of brain metastasis and primary disease. We attempted to account for this by incorporating measures correlating with illness severity, such as age and comorbidity status. More broadly characterizing the impact of clinical decision-making would require multi-institutional collaborations spanning hospital organizations, data-use agreements, and institutional review board approval. In addition, we only captured inpatient and emergency department data, because outpatient visits are not available through HCUP. This results in 2 additional major limitations of this study. First, the results of this study are more applicable to patients with brain metastasis requiring frequent hospitalizations, rather than those with more stable disease managed in the outpatient setting. Second, mortality rates reported in this study might be biased, although previous studies have shown that approximately 40% of deaths due to brain metastasis occur in the inpatient setting.^16^ Although we cannot directly investigate the rate of outpatient mortality among this patient population, we found that that hospitals of lower connectedness are also more likely to discharge patients to hospice care compared with highly connected hospitals. This suggests that lower connectivity hospitals do not necessarily lack access to hospice services, which could otherwise confound the observed higher inpatient mortality rates in this population.

## Conclusions

A network analysis of brain metastases care demonstrated that increased hospital connectedness was associated with reduced risks of inpatient mortality and reduced LOS. Well-connected hospitals were not always the largest or most resourced facilities, indicating an independent value of hospital connectedness to evaluate hospital quality over traditional metrics. We envision that active efforts to strengthen connections between regional facilities and across integrated delivery networks will ultimately help improve access, quality of care, and outcomes for complex oncological patients of all socioeconomic backgrounds.

## Notes

Supplement 1. **eFigure 1.** Direction of Net Patient Flow Not Associated With Changes in Inpatient Mortality or Length of Stay **eFigure 2.** Percentage of Disposition to Hospice by Hospital Connectivity Quartile

Supplement 2.
Data Sharing Statement
