# Error in Figure

**DOI:** 10.1001/jamanetworkopen.2024.46682

**Published:** 2024-10-31

In the Original Investigation titled “Role of Hospital Connectedness in Brain Metastasis Outcomes,”^1^ published September 23, 2024, there was an error in Figure 3. In panel A, centrality should have been labeled as connectedness and weight should have been labeled as volume. This article has been corrected.^1^
