# Supplementary material for: Role of Hospital Connectedness in Brain Metastasis Outcomes
Source: JAMA Netw Open. 2024 Sep 23;7(9):e2435051. doi: 10.1001/jamanetworkopen.2024.35051 (PMC11420690; doi:10.1001/jamanetworkopen.2024.35051)
Supplement: Supplement 1. — eFigure 1. Direction of Net Patient Flow Not Associated With Changes in Inpatient Mortality or Length of Stay eFigure 2. Percentage of Disposition to Hospice by Hospital Connectivity Quartile [file jamanetwopen-e2435051-s001.pdf]

Supplemental Online Content

Tong L, Patel RV, Aizer AA, Dhand A, Bi WL. Role of hospital connectedness in brain metastasis outcomes. *JAMA Netw Open*. 2024;7(9):e2435051. doi:10.1001/jamanetworkopen.2024.35051

eFigure 1. Direction of Net Patient Flow Not Associated With Changes in Inpatient Mortality or Length of Stay

eFigure 2. Percentage of Disposition to Hospice by Hospital Connectivity Quartile

This supplemental material has been provided by the authors to give readers additional information about their work.

© 2024 Tong L et al. *JAMA Network Open*.

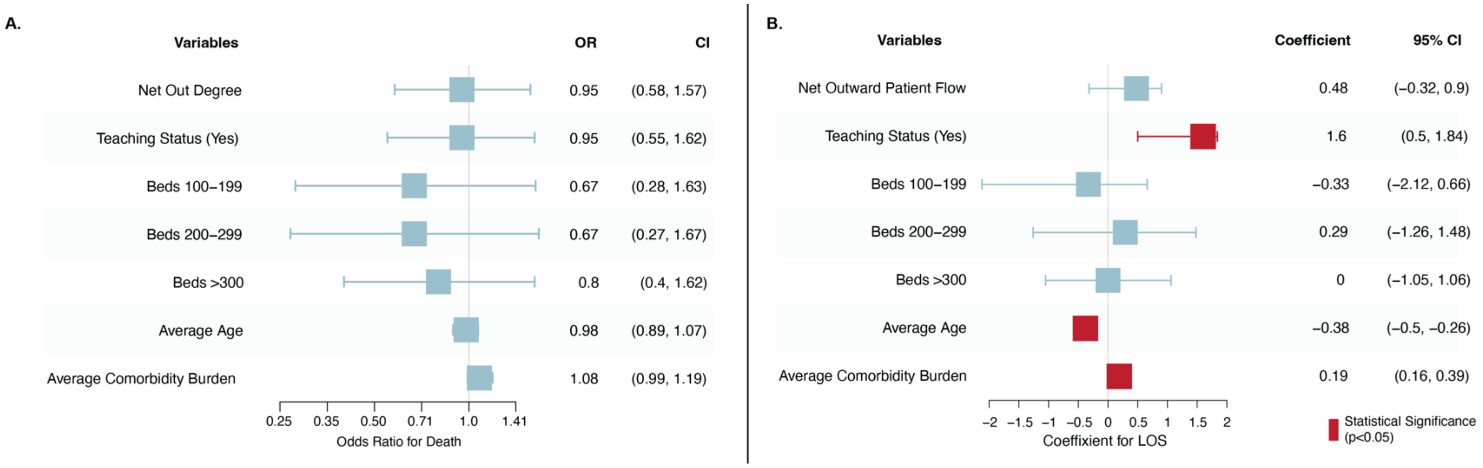

eFigure 1. Direction of Net Patient Flow Not Associated With Changes in Inpatient Mortality or Length of Stay. The association of direction of net patient flow (outward vs. inward [ref]) with (A) rate of inpatient mortality and (B) length of stay, adjusted for teaching status, bedsize, average age, and average comorbidity burden. (calculated by Charlson Comorbidity Index). OR, odds ratio; CCI, Charlson Comorbidity Index; CI, confidence interval

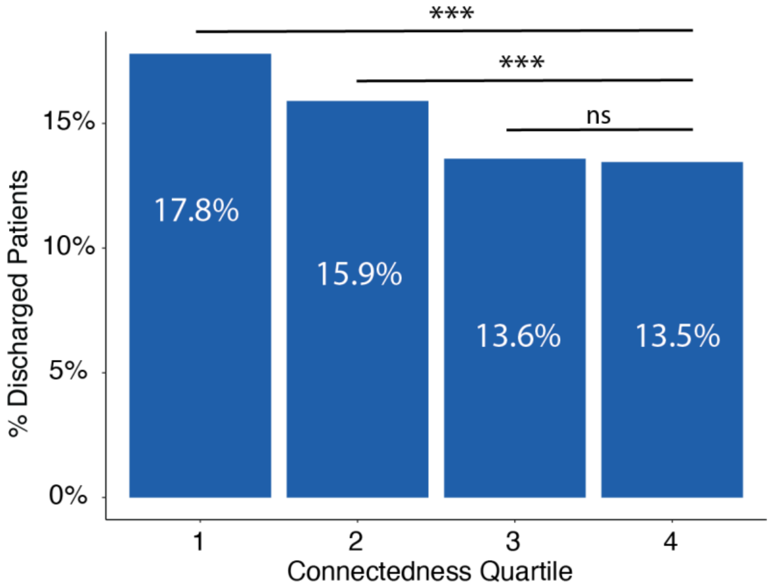

eFigure 2. Percentage of Disposition to Hospice by Hospital Connectivity Quartile. Rate of disposition to hospice (either home or medical hospice facility) calculated for hospitals of each connectivity quartile. ***: p<0.001

© 2024 Tong L et al. *JAMA Network Open*.
