# Supplementary material for: Role of Hospital Connectedness in Brain Metastasis Outcomes
Source: JAMA Netw Open. 2024 Sep 23;7(9):e2435051. doi: 10.1001/jamanetworkopen.2024.35051 (PMC11420690; doi:10.1001/jamanetworkopen.2024.35051)
Supplement: Supplement 2. — Data Sharing Statement [file jamanetwopen-e2435051-s002.pdf]

Data Sharing Statement

Tong. Role of Hospital Connectedness in Brain Metastasis Outcomes. *JAMA Netw Open*. Published September 23, 2024. doi:10.1001/jamanetworkopen.2024.35051

Data
Data available: No

Additional Information
Explanation for why data not available: Data available via HCUP through data usage approval.
